# Three-Dimensional Poly-(ε-Caprolactone) Nanofibrous Scaffolds Promote the Maturation of Human Pluripotent Stem Cells-Induced Cardiomyocytes

**DOI:** 10.3389/fcell.2022.875278

**Published:** 2022-08-01

**Authors:** Mingming Zhang, Yuerong Xu, Yan Chen, Qinru Yan, Xiaoli Li, Lu Ding, Ting Wei, Di Zeng

**Affiliations:** ^1^ Department of Cardiology, Tangdu Hospital, the Fourth Military Medical University, Xi’an, China; ^2^ Department of Orthodontics, School of Stomatology, the Fourth Military Medical University, Xi’an, China; ^3^ Department of Cardiology, 971th Hospital, Chinese People’s Liberation Army Navy, Qingdao, China; ^4^ Department of Neurological Rehabilitation, Xi ‘an International Medical Center Hospital, Xi’an, China

**Keywords:** nanofibrous scaffolds, electrospinning, cardiomyocytes maturation, extracellular matrix, induced pluripotent stem cell

## Abstract

Although pluripotent stem cell-derived cardiomyocytes (iPSC-CMs) have been proved to be a new platform for heart regeneration, the lack of maturity significantly hinders the clinic application. Recent researches indicate that the function of stem cell is associated with the nanoscale geometry/topography of the extracellular matrix (ECM). However, the effects of 3D nanofibrous scaffolds in maturation of iPSC-CMs still remain unclear. Thus, we explored the effects of restructuring iPSC-CMs in 3D nano-scaffolds on cell morphology, cardiac-specific structural protein, gap junction and calcium transient kinetics. Using the electrospinning technology, poly-(ε-caprolactone) (PCL) nanofibrous scaffold were constructed and iPSC-CMs were seeded into these forms. As expected, strong sarcolemmal remodeling processes and myofilament reorientation were observed in 3D nano-scaffolds culture, as well as more expression of cardiac mature proteins, such as β-MHC and MLC2v. The mature morphology of 3D-shaped iPSC-CMs leaded to enhanced calcium transient kinetics, with increased calcium peak transient amplitude and the maximum upstroke velocity (Vmax). The results revealed that the maturation of iPSC-CMs was enhanced by the electrospun 3D PCL nanofibrous scaffolds treatment. These findings also proposed a feasible strategy to improve the myocardium bioengineering by combining stem cells with scaffolds.

## Introduction

iPSC-CMs have enormous potential for the heart regeneration and drug screening in clinic. The differentiation efficiency had been improved in recent years (Karbassi et al., 2020a). However, the morphology, physiological function and electrophysiological characteristics of iPSC-CMs are still immature compared to adult cardiomyocytes (CMs). The iPSC-CM are composed of immature ventricles, atria, and pacemaker cells, which are not suitable for heart disease modeling and drug screening in specific cardiomyocyte subtypes. Moreover, there are potential safety risk of teratoma and arrhythmia after *in vivo* transplantation of iPSC-CMs due to the poor functional mecha-electrical coupling between iPSC-CMs and *in vivo* cardiomyocytes (Liu et al., 2018). Therefore, it is urgent to explore a more applicable strategy for iPSC-CM maturation, which is closer to the electrophysiological and mechanical properties of adult cardiomyocytes.

Numerous approaches to promote iPSC-CMs maturation have been explored, including prolonging the time of cell culture (Sartiani et al., 2017), metabolic hormonal treatment (Parikh et al., 2017), substrate stiffness (Feaster et al., 2015), and tissue engineering (Ronaldson-Bouchard et al., 2018). However, it is still necessary to improve the iPSC-CMs maturation because of the insufficient maturation based on these approaches. The maturation of cardiomyocytes is a complex process, which is unlikely to be dominated and controlled by a single pathway. For this reason, the combination of multiple methods to better mimic the maturation microenvironment of natural myocardium is expected to promote the maturation of iPSC-CMs. Currently, ECM is the most ideal appropriate combination carrier, since it can shape different biomechanical properties using various methods such as matrix stiffness modulation, micro-patterning and 3D culture (Herron et al., 2016) to affect the maturation of cardiomyocytes (Huang et al., 2020). Our previous study also found that the biomimetic microenvironment established by 3D matrix culture can promote the directed differentiation of iPSC-CMs (Chen et al., 2015). However, the effect of mimicked ECM microenvironment by nano-matrix scaffolds on the maturation of iPSC-CMs is still unclear. In this study, we prepared ECM nano-matrix scaffolds using electrospinning technology to mimic the natural myocardial ECM microenvironment, and explored its effect on the maturation of iPSC-CMs.

## Materials and Methods

### Cell Culture

The iPSCs carrying the GFP transgene targeted to the Oct4 locus (kindly provided by Duanqing Pei, Chinese Academy of Sciences) were cultured with basic fibroblast growth factor (Chemicon, Temecula, CA). CMs were cultured as previously report (Zeng et al., 2013). The iPSCs were planted on the electrospun 3D PCL at the beginning of the induction differentiation. Undifferentiated human iPSCs were cultured on a mitotically inactivated mouse embryonic fibroblast (MEF; 50,000 cells/cm2) feeder layer or on gelatin-coated dishes. Starting at day 2 of differentiation, the differentiation medium was replaced every second day.

### Nanofibrous Scaffold Preparation by Electrospinning

**FIGURE 1:**
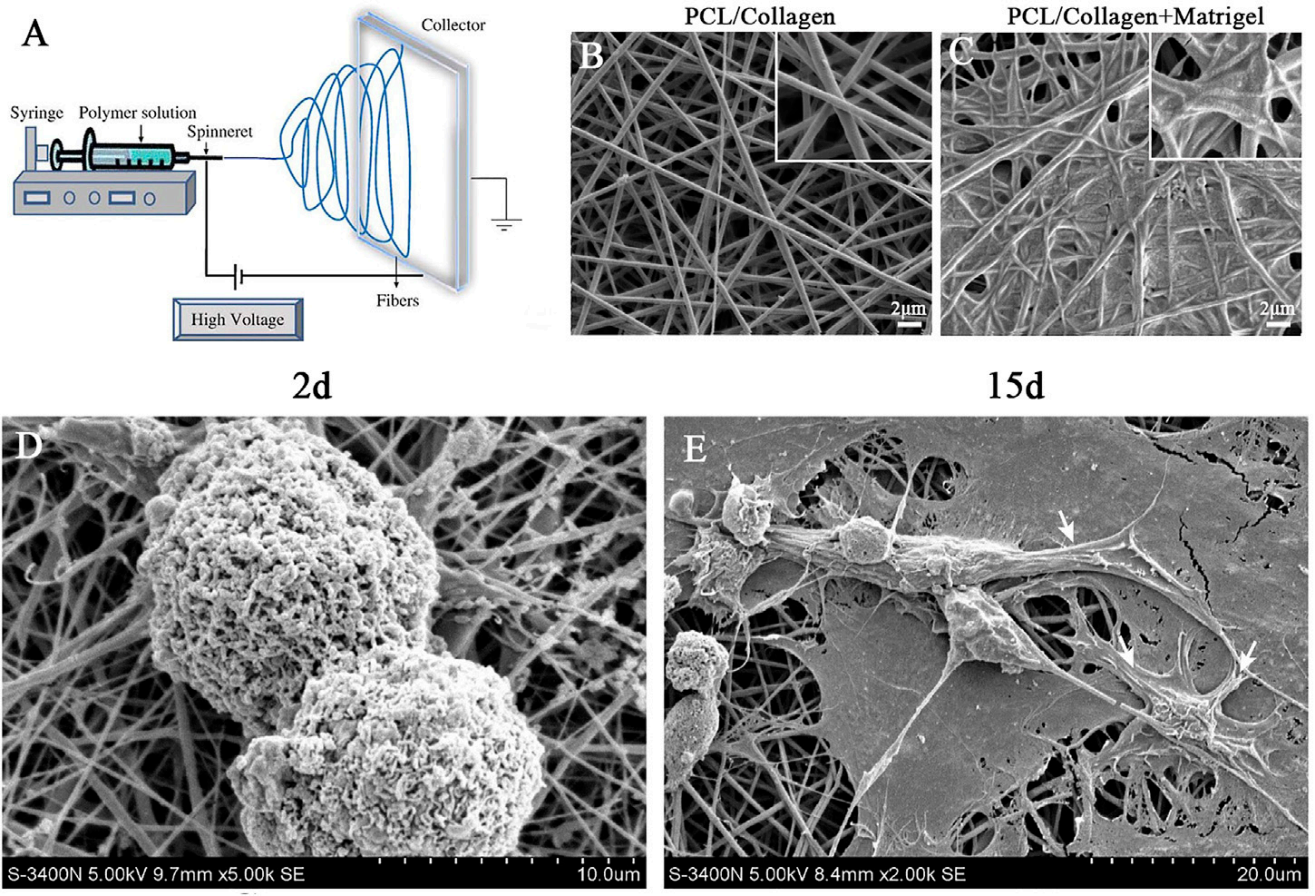
Electrospinning was used to prepare and characterize nanofibrous scaffold. **(A)** Schematic diagram of electrospinning technology. **(B)** SEM micrographs of PCL/collagen nano-scaffold without matrigel coating. **(C)** SEM micrographs of PCL/collagen nano-scaffold with matrigel coating. **(D)** SEM micrographs of iPSCs colonies cultured on nano-scaffold at day 2. **(E)** SEM micrographs of iPSC-CMs colonies cultured on nano-scaffold at day 15. White arrows indicate CM-like morphology. Scale bar in **(B–C)**: 2 μm.

The 3D poly-(ε-caprolactone) (PCL) nanofibrous scaffold was fabricated using electrospinning technology based on our previously study (Chen et al., 2015). The schematic diagram was shown in Figure 1A. The 3D PCL nanofibrous scaffold was fabricated by electrospinning. Briefly, pellets of PCL with an average molecular weight of 80 kDa (Aldrich, St. Louis,MO, United States) were dissolved in a chloroform/methanol (3:1, v/v) solvent mixture at room temperature (25 ± 1°C) to obtain a 20 wt% solution. Then, the PCL solution was placed in a 10-ml plastic syringe connected to a bluntstainless steel needle with an inner tip diameter of 0.5 mm. A syringe pump (PHD22/2000, Harvard Apparatus, Holliston, MA, United States) was used to control the flow rate at 2 ml/h. A 20-kV high-voltage power supply and a distance of approximately 15 cm were maintained between the needle and the grounded flat aluminum plate collector (size: 15 × 15 cm^2^) throughout the electrospinning process. Then, the PCL fibrous scaffolds were sterilized under ultraviolet (UV) light for 1 h. Finally, the scaffold was precoated with gelatin (Sigma-Aldrich, St. Louis, MO, United States) by immersion in a sterilized 0.1% (1 g/l) gelatin solution overnight.

### Scanning Electron Microscopy

The morphology of electrospun PCL fibrous scaffolds was observed by SEM as previously described (Chen et al., 2015).

### Immunocytochemistry

After differentiated for 15 days, the cells were fixed and incubated with primary antibodies (anti-MLC-2V, anti-α-actinin, anti-Ki67, anti-MHC, anti-connexin 43 (Cx43) and anti-cardiac troponin T (cTnT), 1:200, all from Abcam, Cambridge, MA, United States) for 60min at room temperature.

### Morphological Analysis

The cell size and sarcomere length were analyzed using ImageJ analysis software.

### Calcium Imaging

The ratiometric indicator dye Fura-4 AM was applied to measure intracellular calcium.

### qRT-PCR

Total RNA was extracted using Trizol reagent (Invitrogen, Carlsbad, CA, United States). PCR was performed as previously described (Chen et al., 2015).

### Western Blotting

Total protein was extracted from cells underwent differentiation for 15 days. The primary antibodies as follows: α-MHC,β-MHC (1:1,000; Abcam, Cambridge, MA, United States), MLC2a, MLC2v (1:1,000, Santa Cruz Biotechnology, Dallas, TX, United States) and GAPDH (1:10,000; Abcam, Cambridge, MA, United States). The band intensities were quantified using ImageJ software.

### Statistical Analysis

Results are presented as mean ± SEM. Data were analyzed by Student’s t test or one-way ANOVA with repeated-measures analysis. *p* < 0.05 were considered statistically significant.

## Results

### Characterization of Nanofibrous Scaffolds

As shown in Figure 1A, nanofibrous scaffolds were prepared with PCL/collagen solutions using electrospinning. SEM of nanofibrous scaffolds revealed cross arrangement of nanofibers without beads, which formed a porous 3D structure (Figure 1B). The nanofibrous scaffolds were pre-coated with matrigel for iPSCs adhension. SEM of nanofibrous scaffolds pre-coated with matrigel revealed that the nanofibers are bridged by matrigel but remained many interconnected pores (Figure 1C). This porous 3D structure was essential for oxygen and nutrition exchange during the CM differentiation of iPSCs.

Moreover, colonies of iPSCs were scattered and adhered well to the nanofibrous scaffolds during day 2 of CM differentiation (Figure 1D). At day 15 of CM differentiation, SEM revealed that the iPSCs spontaneously differentiated into many cells with morphology similar to neonatal cardiomyocytes (NCMs, Figure 1E). Based on above results, we conclued that the nanofibrous scaffold was a suitable three-dimensional carrier for the differentiation of iPSCs into CMs.

### Spontaneous Cardiac Differentiation of iPSCs on 3D Nanofibrous Scaffolds

**FIGURE 2:**
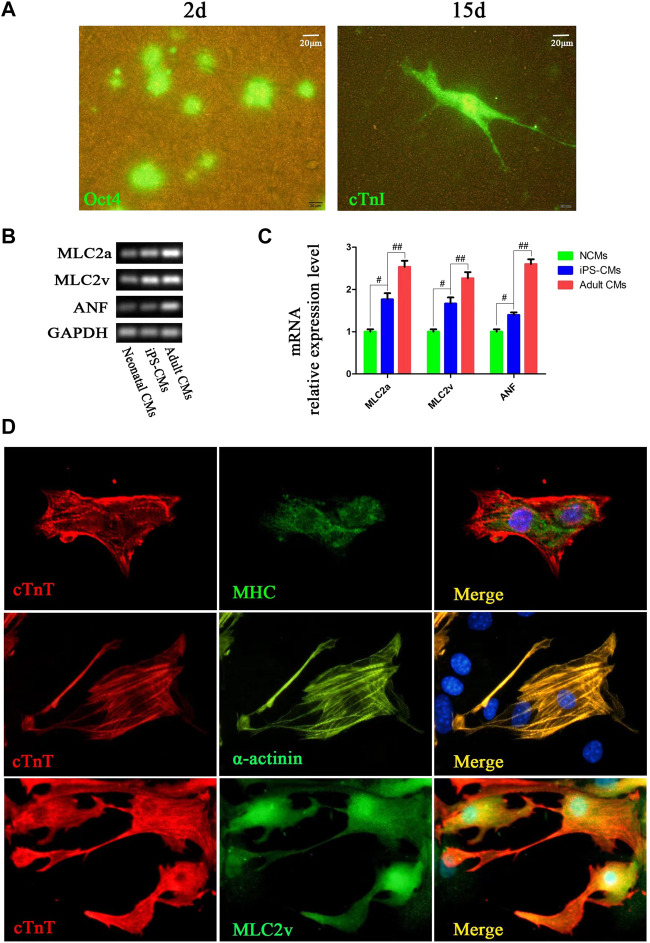
CMs was differentiated from hPSCs cultured on 3D nanofibrous scaffold. **(A)** Fluorescence expression of Oct4 (day 2) and cTnI (day 15) in differentiation. **(B,C)** RT-PCR analysis of cardiac-specific gene expression in differentiated iPSC-CMs cultured with 3D nano-scaffolds, NCMs and adult CMs. **(D)** Immunofluorescence co-staining of cTnT with MHC, α-actinin and MLC2v in cells at day 15 of differentiation on 3D nanofibrous scaffolds to identify the CM phenotype.

After the purified Oct4-GFP^+^ iPSCs without MEFs were seeded on the 3D nanofibrous scaffold, the expression of Oct4 was decreased during day 2 of differentiation (Figure 2A). Cells with morphology similar to neonatal cardiomyocytes were differentiated during day 15 of differentiation, and the immunofluorescence for cTnT indicated that the differentiated cells were iPSC-CMs. Meanwhile, the gene markers for detecting the phenotype of CM differentiation was assessed compared to NCMs and adult CMs. The expression of ANF, MLC2a and MLC2v of iPSC-CMs cultured on 3D nanofibrous scaffolds were significantly higher than that of NCMs, but slightly lower than that of adult CMs (Figure 2B). Moreover, double staining of the cTnT with MHC, α-actinin, and MLC2v showed that the iPSC-CMs with the scaffolds co-expressed the CM phenotype markers, including cTnT with MHC, α-actinin, and MLC2v (Figure 2C). Thus, the 3D nanofibrous scaffold could promote the differentiation of iPSCs into functional CMs.

### Nanofibrous Scaffolds Enhance Structural Maturation of iPSC-CMs

**FIGURE 3:**
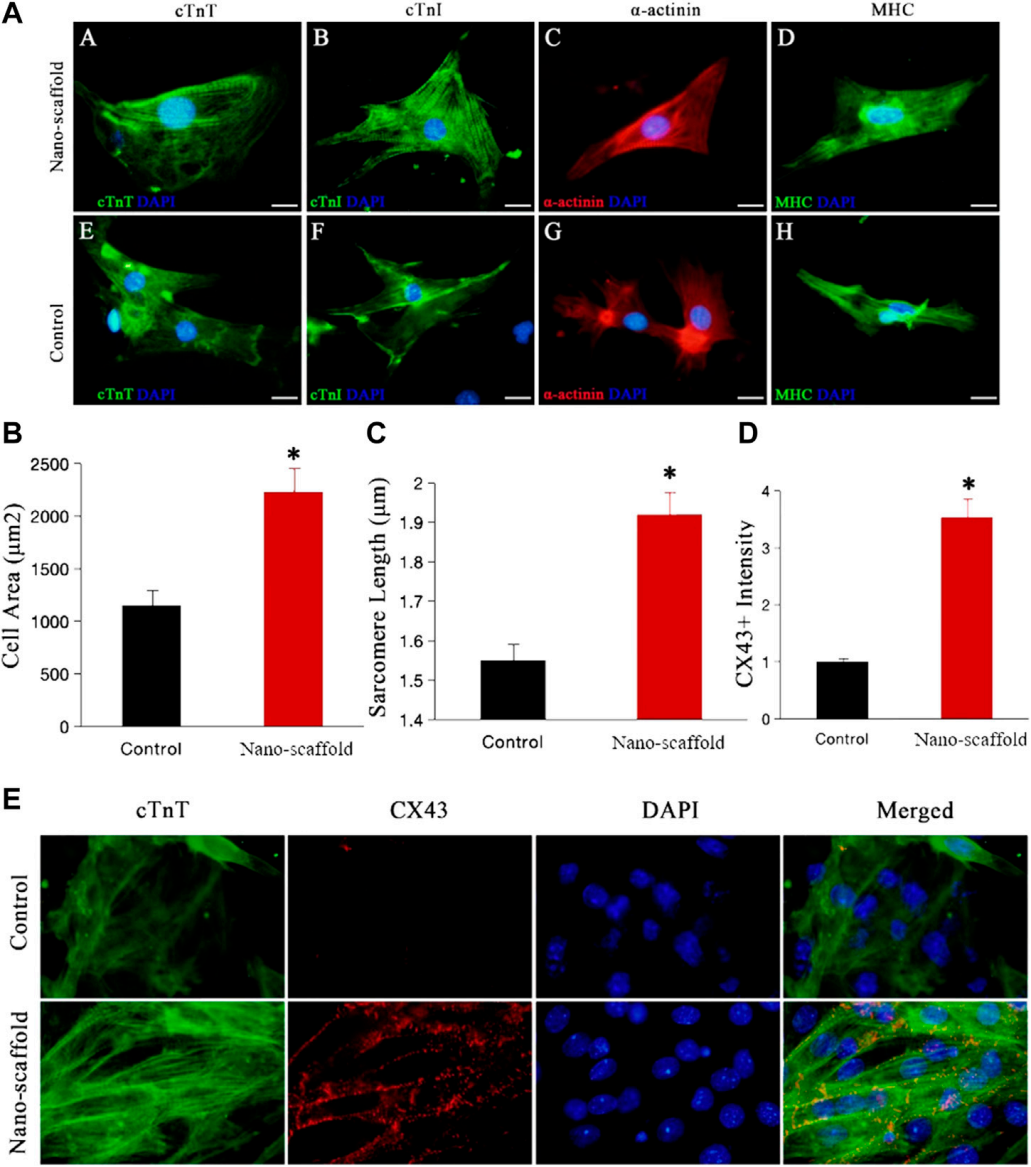
Confocal microscopy was used to evaluate the structural maturation of iPSC-CMs cultured on nanofibrous scaffold. **(A)** Fluorescence expression of cardiac-specific structural protein (cTnI, MHC, a-actinin and cTnT) in both control and nano-scaffold groups at day 15 of differentiation. Compared with the control group, iPSC-CMs cultured on nanofibrous scaffold exhibited significant changes in cell area **(B)** and sarcomere length **(C)**. **(D,E)** Immunofluorescence co-staining of cTnT with gap junction protein Cx43 in control and nano-scaffold groups and the Cx43^+^ fluorescence expression was quantified. Data are presented as mean ± SEM, *p < 0.05 vs. control.

To clarify the effects of nanofibrous scaffolds on the iPSC-CMs, we detected the cardiac-specific structural protein such as MHC, a-actinin, cTnT (a sarcomere protein) and MLC-2v (a key protein for structural maturation) (Karbassi et al., 2020b). iPSC-CMs were small in shape with disordered myofibril arrangement in control group, while iPSC-CMs formed well-organized sarcomeric myofilaments in cytoplasmic patterns on nano-scaffolds (Figure 3A). Measurement of cell area showed that 3D nano-scaffolds culture increased the cell area by 90% (1,153 ± 145 μm^2^ vs. 2,235 ± 207 μm^2^, *p* < 0.01), indicating a more mature cardiomyocyte phenotype (Figure 3B). Moreover, sarcomere length of iPSC-CMs was increased from 1.55 ± 0.32 μm in control to 1.92 ± 0.28 μm in iPSC-CMs cultured on nano-scaffolds (*p* < 0.05) (Figure 3C). The gap junctions between CMs and neighbor cells are critical to the maturation of cardiomyocytes. We subsequently explored the effect of 3D nano-scaffolds on expression of gap junction protein Cx43. The immunofluorescence co-staining of Cx43 and cTnT showed that Cx43 expression was significantly higher in iPSC-CMs cultured on nano-scaffolds than that in the control group (Figures 3D,E). These results indicated that nanofibrous scaffolds enhances structural maturation of iPSC-CMs with mature sarcomere structures such as Z-line and T tube.

### Nanofibrous Scaffolds Enhance the Expression of Cardiac Mature Proteins

**FIGURE 4:**
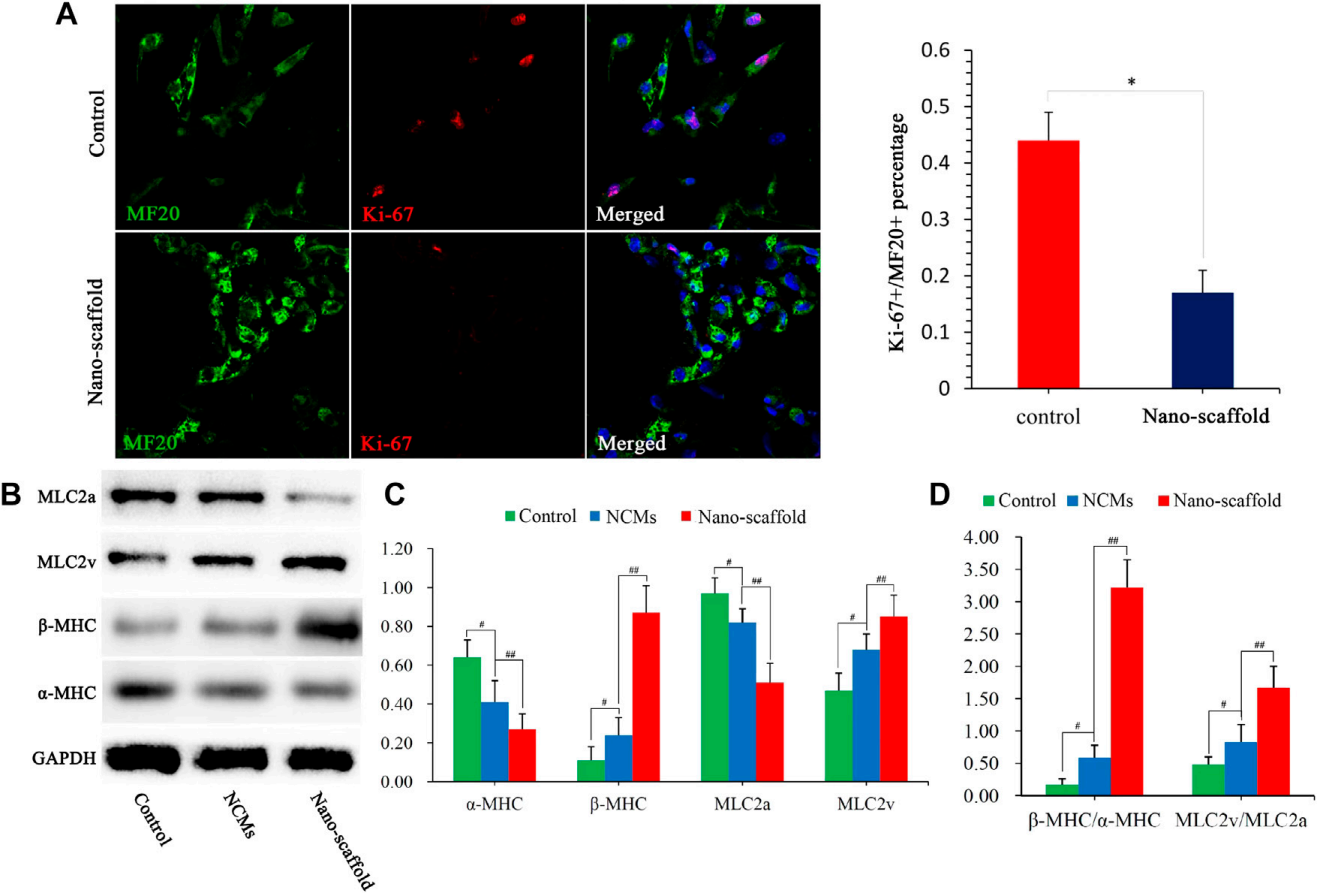
Cardiac mature proteins were analyzed by different assays. **(A)** Co-staining of Ki-67 and MF20 to detect the proliferation ability of iPSC-CMs, and Ki-67^+^/MF20^+^ percentage was quantified. **(B)** Protein expression of α-MHC, β-MHC, MLC2a, and MLC2v were assessed in control, NCMs and nano-scaffold groups. The values are normalized to the housekeeping gene GAPDH. **(C)** Quantification of α-MHC, β-MHC, MLC2a and MLC2v protein levels in NCMs, control and nano-scaffold groups. **(D)** Expression ratio of β-MHC/α-MHC and MLC2v/MLC2a as mature markers in three groups. Expression levers of each gene were normalized to GAPDH. Data are presented as mean ± SEM, #*p* < 0.05 vs. control; ##*p* < 0.05 vs. NCMs.

Mature CMs are not capable to proliferate, so the proliferation ability can be used as an indirect indicator of CMs maturation. Co-staining of proliferating cell nuclear antigen Ki-67 and sarcomeric myosin MF20 showed that the Ki-67^+^/MF20^+^ percentage was lower in iPSC-CMs cultured on nano-scaffolds than that in iPSC-CMs in control group (Figure 4A), suggesting more mature CM phenotype with 3D nano-scaffold culture. The sarcomere-unit components are changed as the heart develops. For example, α-MHC at fetal stage switches to β-MHC at adult stage and MLC2v expression is also considered to be a marker of CM maturity. To further confirm the mature phenotype of iPSC-CMs, the expression of α-MHC, β-MHC, MLC2a and MLC2v were detected. The expression levels of β-MHC and MLC2v were higher in nano-scaffold group than that in the control group and NCMs, while the expression levels of α-MHC and MLC2a were significantly reduced (Figures 4B,C). Moreover, the ratio of β-MHC/α-MHC and MLC2v/MLC2a were increased when iPSC-CMs were cultured on nano-scaffolds. Both of the increased expression of cardiac mature proteins and reduced proliferation capacity indicated that 3D nano-scaffold culture enhanced the maturation of CMs derived from human PSCs (hPSCs).

### Nanofibrous Scaffolds Enhance Calcium Transient Kinetics of iPSC-CMs

**FIGURE 5:**
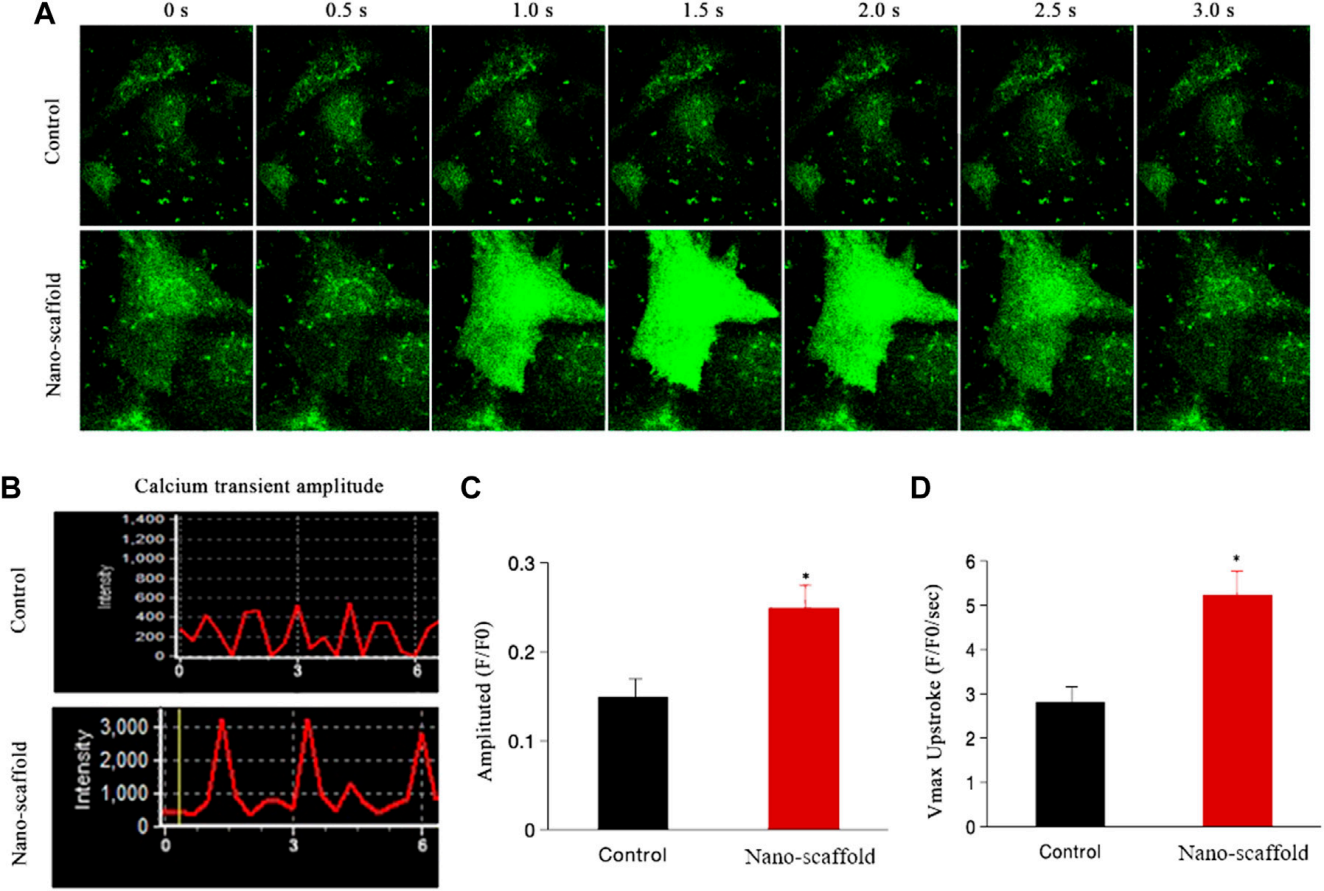
Calcium transient was assessed in iPSC-CMs cultured on nanofibrous scaffold. **(A)** Representative line-scan of time-lapse calcium imaging in iPSC-CMs loaded with the intracellular calcium indicator Fluo-4 AM. **(B)** Representative records of spontaneous calcium transient. **(C)** Comparison of calcium transient peak amplitude between two groups. **(D)** Comparison of Vmax (F/F0/s) between two groups. Data are presented as mean ± SEM, *p < 0.05 vs. control.

We detected the intracellular ratiometric calcium dye Fura-4 AM to compare the calcium transient kinetics in control versus iPSC-CMs cultured on nano-scaffolds under 1-Hz electrical stimulation. The intracellular calcium transient was recorded (Figure 5A). The calcium peak transient amplitude of iPSC-CMs was increased by nano-scaffolds culture was increased by nano-scaffolds culture (0.15 ± 0.03vs. 0.25 ± 0.04, F/F0, *p* < 0.05, Figures 5B,C). The maximal upstroke velocities were also higher in iPSC-CMs cultured on nanofibrous scaffolds. Specifically, Vmax increased more rapidly in nano-scaffolds group (2.81 ± 0.42 vs. 5.24 ± 0.53 F/F0/s, *p* < 0.05, Figure 5D). These results indicated that iPSC-CMs exhibited the enhanced CM-specific calcium handling properties and functional maturation when cultured on nanofibrous scaffolds.

## Discussion

iPSC-CMs are the ideal cardiomyocyte source for replacing dysfunctional CMs in the future because of the unique electrophysiological and distinctive contractile properties (Mummery, 2018). However, even after differentiation, iPSC-CMs retain a part of immature phenotype. There is still no better way to make these cells more mature to achieve the complicated structural and functional phenotype of CMs *in vitro* (Zhu et al., 2014). Thus, increased attempts have been made to optimize the maturation of hPSC-CMs via diverse approaches (Mummery, 2018; Yang et al., 2019). However, the early efforts to mature these cells by implanting them in the heart have been still insufficient (Shiba et al., 2012; Chong et al., 2014). 3D nano-scaffold culture has been recommended as one of the methods to optimize the differentiation efficiency of iPSC-CMs, which had been proved to promote the CM differentiation from iPSCs by our previous studies (Ou et al., 2013). However, it is still unclear whether 3D nano-scaffold culture is enough to reinforce the robust maturation of hPSC-CMs. In this research, we synthesized nanofibrous scaffolds with PCL/collage components to simulate the matrix microenvironment of myocardial maturation, and evaluated the efficacy of these scaffolds on the maturation of iPSC-CMs. As expected, iPSC-CMs on nanofibrous scaffolds preferentially differentiated to mature CMs. The difference with the cells on TCP is that the iPSC-CMs on nanofibrous scaffolds significantly enhanced structural maturation with increased levels of cardiac-specific proteins and well-organized sarcomeric myofilaments. Moreover, calcium transient kinetics of iPSC-CMs were promoted by nanofibrous scaffolds, proposing that the functional maturation of iPSC-CMs was optimized by nanofibrous scaffolds.

In contrast to adult CMs, iPSC-CMs under normal culture fail to generate the characteristic sarcolemma invaginations (Gherghiceanu et al., 2011). The absence of the regular cell structure with disorganized distribution of myofibrils leads to discordant contraction. Differentiation and growth of iPSC-CM monolayers is prevalent due to the simplicity and moderate scalability of this approach for CM generation. The heart is a 3D organ structure which favors the development of the heart because CMs can remain natural contact with neighboring cells. However, the extracellular matrix is improbable to occur in the 2D environment of standard cell culture (Kshitiz Park et al., 2012). As a result, more and more investigators optimized the *in vitro* microenvironment to better reproduce the *in vivo* myocardium environment. The biophysical features of fibrous ECM, for example, its geometric/topographical characteristics at the nanoscale level and the transmission of biophysical factors to the cell, are vital for cardiomyocyte function and destiny decisions (Huebsch et al., 2010; Zuppinger, 2016). Our previous studies had proven the advantages of culturing iPSC-CMs in 3D systems (fibrous ECM) to enhance the differentiation efficiency of iPSC-CMs (Chen et al., 2015). Fibrous ECM nano-scaffolds which provide not only the ECM microenvironment but also 3D architecture for cardiac development. We hypothesized that the fibrous ECM nano-scaffolds could facilitate iPSC-CMs towards more mature phenotype. Previous studies reported that polymeric scaffolds comprising different ECM components promoted the differentiation of hiPSCs to functional CMs (Gupta et al., 2011; Lemoine et al., 2017; Ulmer et al., 2018). However, cells were pretreated with Noggin in previous study, which made it difficult to decipher the cardiogenesis effect on these scaffolds. To validate the direct effect of 3D nanofibrous scaffolds on the maturation of CM derived from iPSCs, we directly seeded iPSCs on nanofibrous scaffolds without any additional factors or modulators through the monolayer approach. In our research, the biophysical signals of the 3D nanofibrous scaffolds could facilitate the structural maturation of iPSC-CMs, including the morphology, sarcomeres, and gap junction.

A characteristic sign of iPSC-CMs immaturity is their spontaneous beating behavior resulting from spontaneous depolarizations and contraction due to calcium transients. Mature cardiomyocyte morphology not only supplies the constructure framework but also directly sets other key functional properties such as electrophysiology and contractility. Therefore, we hypothesized that fibrous ECM nano-scaffolds could also enhance the functional maturation due to the enhancement effect on structural maturation of iPSC-CMs. The exact underlying mechanisms of automaticity and contractility may include immature calcium handling, which might be relevant to the expression of premature protein isoforms which were involved in regulation of calcium homeostasis (Lipskaia et al., 2014). Our results revealed that the calcium peak transient amplitude and Vmax of iPSC-CMs were increased by 3D nanofibrous scaffolds, which indicated more mature calcium transient kinetics. Our data also demonstrated that the induced elongated cell shape had a dramatic effect on cell microstructure, distribution patterns, membrane properties, and protein expression, which resulted in strong calcium handling during spontaneous and triggered activity. Therefore, we suggest that tight control of 3D architecture can induce structural and functional maturation development in iPSC-CMs.

The elaboration of maturation mechanisms in CM remain incomplete. In addition, an unified definition of maturation is still absent. Regarding cardiac development, multiple factors including biophysical, biochemical or biological cues may influence cardiomyocyte maturation. Accordingly, previous studies have involved molecular targets, genetic manipulation methods, and cell co-culture or implantation to induce further maturation of iPSC-CMs, especially in structural or functional properties (Abilez et al., 2017; Sun and Nunes, 2017). However, these strategies are difficult to better recapitulate the *in vivo* myocardium environment. There are still some challenges and difficulties in facilitating the maturation of iPSC-CMs *in vitro*. First, iPSC-CMs are considerably distinguished from adult human ventricular CMs, as shown in the present study. For instance, the sarcomere length of relaxed human cardiac muscle cell is about 2.8 μm (Bird et al., 2003), which is approximately 1.5 times the value of hPSC-CMs observed in this study. It can take 6,7 years for human CMs to achieve some of the characteristics of adult CMs *in vivo* (Peters et al., 1994), indicating the essential need of accelerated maturation for iPSC-CMs differentiated *in vitro*. Secondly, most studies so far, including our present research, have used heterogeneous iPSC-CM population. Heterogeneous maturation levels include immature embryonic or neonatal-like CMs, which are refiected in different levels of maturation in sarcomeric organizations and electrophysiological characteristics (Yang et al., 2014; Veerman et al., 2015). We speculated that our electrospun fibers prepared with PCL/collagen and matrigel could recapitulate 3D nano-scaffolding environment for cardiac development and maturation. In future studies, the fiber size/scaffold topology can be optimized by modulating the electrospinning parameters (Han et al., 2013) to facilitate the cell spreading and maturation (Kuroda et al., 2014). In addition, a stage-specific dynamic design of the fiber size/scaffold topology may be more advantageous, as iPSC-CMs constantly enlarge during maturation.

## Conclusion

In summary, our study confirmed that the artificial 3D nanofibrous scaffold is an excellent substrate for the maturation of iPSC-CMs. The study also identified that 3D nanofibrous scaffolds could directly facilitate the structural and functional maturation of iPSC-CMs, which was arguably an optimal method to promote maturation *in vitro*. Our findings presented new perspectives on novel biomimetic scaffolds to guide the efficient maturation of iPSCs for future myocardial tissue engineering and regenerative medicine.

## Data Availability

The original contributions presented in the study are included in the article/Supplementary Material, further inquiries can be directed to the corresponding author.
